# Bulked Segregant Analysis Coupled with Whole-Genome Sequencing (BSA-Seq) Mapping Identifies a Novel *pi21* Haplotype Conferring Basal Resistance to Rice Blast Disease

**DOI:** 10.3390/ijms21062162

**Published:** 2020-03-21

**Authors:** Tingmin Liang, Wenchao Chi, Likun Huang, Mengyu Qu, Shubiao Zhang, Zi-Qiang Chen, Zai-Jie Chen, Dagang Tian, Yijie Gui, Xiaofeng Chen, Zonghua Wang, Weiqi Tang, Songbiao Chen

**Affiliations:** 1 Marine and Agricultural Biotechnology Laboratory, Institute of Oceanography, Minjiang University, Fuzhou 350108, China; LTM0521@163.com (T.L.); chiwenchao@mju.edu.cn (W.C.); chenxf@mju.edu.cn (X.C.); wangzh@fafu.edu.cn (Z.W.); 2 Biotechnology Research Institute, Fujian Academy of Agricultural Sciences, Fuzhou 350003, China; czq@fjage.org (Z.-Q.C.); czj@fjage.org (Z.-J.C.); tdg@fjage.org (D.T.); gyj@fjage.org (Y.G.); 3 College of Agriculture, Fujian Agriculture and Forestry University, Fuzhou 350002, China; likun.huang@foxmail.com (L.H.); zhangsbiao@aliyun.com (S.Z.); 4 State Key Laboratory of Ecological Pest Control for Fujian and Taiwan Crops, Fujian Agriculture and Forestry University, Fuzhou 350002, China

**Keywords:** rice, blast disease, partial resistance, *pi21*, haplotype

## Abstract

Basal or partial resistance has been considered race-non-specific and broad-spectrum. Therefore, the identification of genes or quantitative trait loci (QTLs) conferring basal resistance and germplasm containing them is of significance in breeding crops with durable resistance. In this study, we performed a bulked segregant analysis coupled with whole-genome sequencing (BSA-seq) to identify QTLs controlling basal resistance to blast disease in an F_2_ population derived from two rice varieties, 02428 and LiXinGeng (LXG), which differ significantly in basal resistance to rice blast. Four candidate QTLs, *qBBR-4*, *qBBR-7*, *qBBR-8*, and *qBBR-11*, were mapped on chromosomes 4, 7, 8, and 11, respectively. Allelic and genotypic association analyses identified a novel haplotype of the durable blast resistance gene *pi21* carrying double deletions of 30 bp and 33 bp in 02428 (*pi21-2428*) as a candidate gene of *qBBR-4*. We further assessed haplotypes of *Pi21* in 325 rice accessions, and identified 11 haplotypes among the accessions, of which eight were novel types. While the resistant *pi21* gene was found only in *japonica* before, three Chinese *indica* varieties, ShuHui881, Yong4, and ZhengDa4Hao, were detected carrying the resistant *pi21-2428* allele. The *pi21-2428* allele and *pi21-2428*-containing rice germplasm, thus, provide valuable resources for breeding rice varieties, especially *indica* rice varieties, with durable resistance to blast disease. Our results also lay the foundation for further identification and functional characterization of the other three QTLs to better understand the molecular mechanisms underlying rice basal resistance to blast disease.

## 1. Introduction

Rice is one of the most important staple crops for more than half of the population in the world [1]. Rice blast, caused by the fungus *Magnaporthe oryzae*, is one of the most devastating diseases of rice, causing yield losses of 10%–30% annually [2]. The development and use of resistant varieties appears to be the most economical and environmentally sustainable way to control rice blast [3]. Identification of genes or genetic loci conferring resistance to blast disease could accelerate breeding programs for resistant rice varieties.

Genetically, disease resistance in plants can be categorized into two types, qualitative and quantitative [4,5]. Qualitative resistance is mainly mediated by a single resistance gene (*R* gene), which confers complete but race-specific resistance through the recognition of pathogen effectors [6,7]. *R* gene-mediated resistance has been widely deployed in crop breeding programs. However, *R* gene-mediated resistance is often not durable, as most pathogens are able to rapidly evolve new virulent races lacking the corresponding avirulence effectors to evade recognition by the cognate R protein. Quantitative resistance is mediated by multiple genes or quantitative trait loci (QTLs), providing partial or basal resistance associated with delayed and reduced development of disease lesions [8,9]. Although quantitative resistance has only partial effects, it has been considered race-non-specific and broad-spectrum, and is therefore of particular interest for breeding crops with durable resistance [4,10].

To date, more than 100 rice blast *R* genes have been identified and at least 28 *R* genes have been cloned [11,12]. All the cloned major *R* genes encode nucleotide-binding site leucine-rich repeat (NBS-LRR) proteins, with the exception of *Pid2*, encoding a B-lectin kinase [13], and *Ptr*, encoding an Armadillo repeat protein [14]. Several hundred QTLs associated with blast resistance have been identified [15]. However, only a limited number of QTLs for blast resistance have been cloned [16,17,18,19,20]. The cloned QTLs encode proteins that are diverse in their structure and function: *pi21* encodes a proline-rich protein with loss-of-function deletions [16]; *Pb1* encodes an atypical coiled-coil (CC)-NBS-LRR protein [17]; *Pi35* and *Pi63* encode NBS-LRR proteins [18,19]; whereas *bsr1-d1* encodes a C2H2-type transcription factor with a single nucleotide change in the promoter [20]. These findings indicate that quantitative resistances are controlled by diverse molecular mechanisms.

With the rapid development of next-generation sequencing, approaches based on bulked segregant analysis coupled with whole-genome sequencing (BSA-Seq) have been developed for the mapping of agronomically important loci in rice [21,22,23], including major genes or QTLs responsible for blast resistance [22,24,25]. In the present study, we apply BSA-seq to rapidly map four QTLs, *qBBR-4*, *qBBR-7*, *qBBR-8*, and *qBBR-11*, responsible for basal resistance to blast disease, and identify a novel haplotype of the durable blast resistance gene *pi21* as a candidate gene of *qBBR-4* on chromosome 4 in a *japonica* variety 02428 (*pi21-2428*). While the resistant *pi21* gene was found only in *japonica* before [16], we identify three Chinese *indica* varieties carrying the resistant *pi21-2428* allele in 325 accessions. Therefore, the novel *pi21-2428* allele and the *pi21-2428*-containing rice varieties identified in the present study provide valuable resources for breeding rice varieties, especially *indica* rice, which are durably resistant to blast disease. Our results also lay the foundation for further identification and functional characterization of the other three QTLs for a better understanding of rice basal resistance to blast disease.

## 2. Results

### 2.1. Evaluation of 02428 and LXG in Basal Resistance to Rice Blast Disease

**Figure 1:**
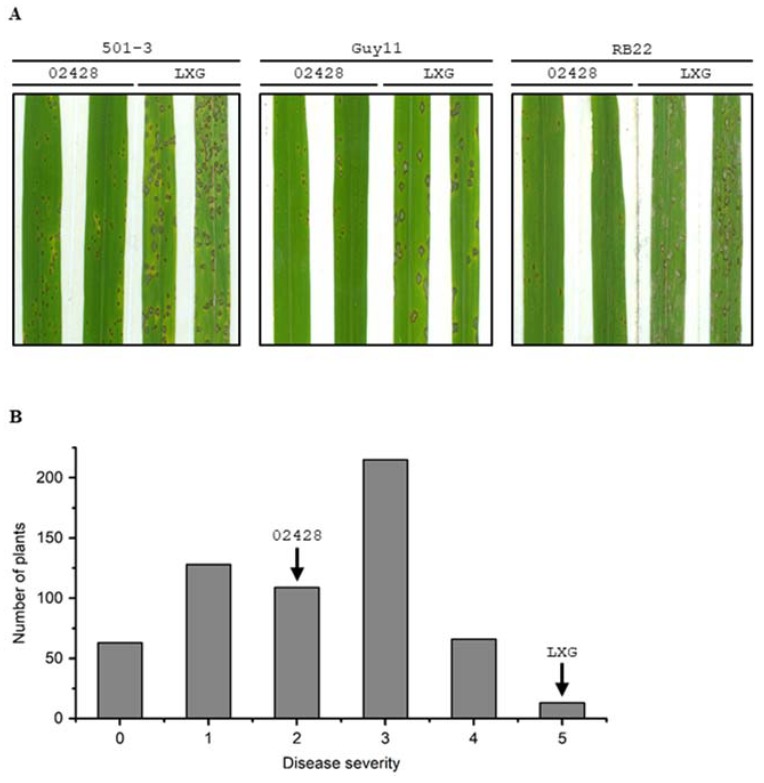
Resistance reaction of rice varieties 02428, LiXinGeng (LXG) and their F_2_ population to rice blast disease. (**A**) Phenotypes of 02428 and LXG inoculated with *M. oryzae* isolates 501-3, Guy11, and RB22. (**B**) The frequency distribution of disease severity in the F_2_ population of 02428 × LXG inoculated with *M. oryzae* isolate RB22. Disease severity was assessed following a 0–5 scale (0–1: resistant, 2: moderately resistant, 3: moderately susceptible, 4–5: severely susceptible).

In our earlier evaluations, the rice variety 02428 was observed to possess high basal resistance to the rice blast fungus *M. oryzae* under natural nursery conditions (data not shown). We further performed artificial inoculations on 02428 seedlings using three virulent isolates of *M. oryzae* in this study. The results showed that 02428 was moderately susceptible to isolates 501-3 and Guy11, and was moderately resistant to isolate RB22 (Figure 1A). Most lesions on leaves of 02428 were limited in size. In contrast, LiXinGeng (LXG) was highly susceptible to all three isolates. These results suggest that 02428 possesses high basal resistance, preventing blast disease development.

An F_2_ population of 02428 × LXG with 626 individuals was inoculated with RB22. The results show that the frequency distribution of disease severity in the F_2_ population of 02428 × LXG exhibited continuous variation (Figure 1B), indicating that the resistance to RB22 is likely controlled by multiple genes.

### 2.2. SNP and Short InDel Polymorphism Profiling

Whole-genome sequencing of extremely resistant (ER) and extremely susceptible (ES) pools derived from the F_2_ population of 02428 × LXG and the two parental lines 02428 and LXG generated about 90.7 to 158.9 million reads for each sub-pool or parental line (Supplementary Table S1). After filtering, a total of 469,512 bi-allelic single-nucleotide polymorphisms (SNPs), and a total of 65,766 bi-allelic short insertions and deletions (InDels) were identified (Table 1). The average densities of SNP and short InDel markers were about 1.26 SNP/kb (average every 795 bp exists a SNP) and 0.18 InDel/kb (average every 5,675 kb exists a short InDel) (Table 1), respectively. The polymorphic markers were sufficiently distributed across the whole genome, except for one region of about 6.5 Mb on chromosome 3 containing relatively fewer markers (Supplementary Figure S1).

### 2.3. QTL Mapping and Heritability Estimation

**Figure 2:**
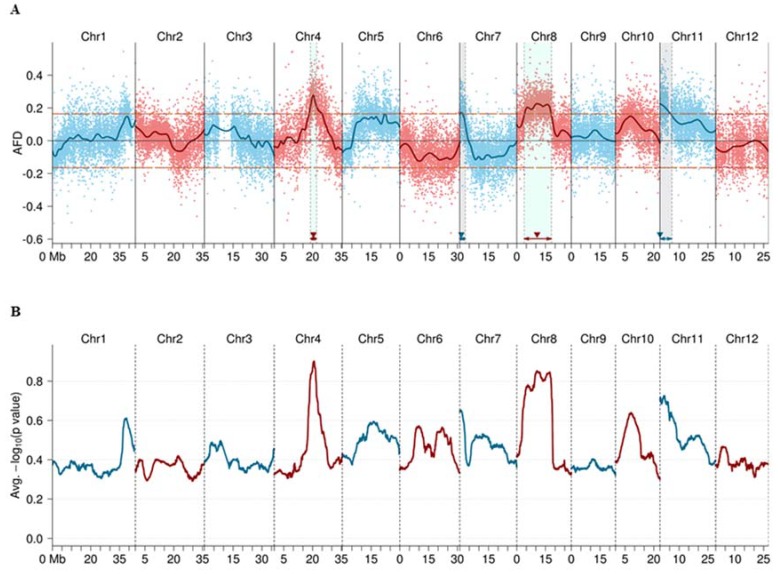
BSA-seq-based identification of four candidate quantitative trait loci (QTLs) conferring basal resistance to rice blast disease. (**A**) Allele frequency difference (AFD) graph from BSA-seq analysis. The horizontal orange dashed lines indicate the threshold (±0.165) at the overall significance level of *p* < 0.05. QTL positions estimated are indicated by filled triangles. AFD was obtained by subtraction of allele frequency (AF) of the extremely susceptible (ES) pool from that of the extremely resistant (ER) pool. (**B**) *T*-test verification for the two replicated sub-pools for the ER pool and the ES pool. The average negative logarithmic *p*-values of the markers were smoothed by sliding window (size = 3000 kb and step = 10 kb) across each chromosome. The peaks of the *p*-value graph were consistent with those in the AFD graph.

**Figure 3:**
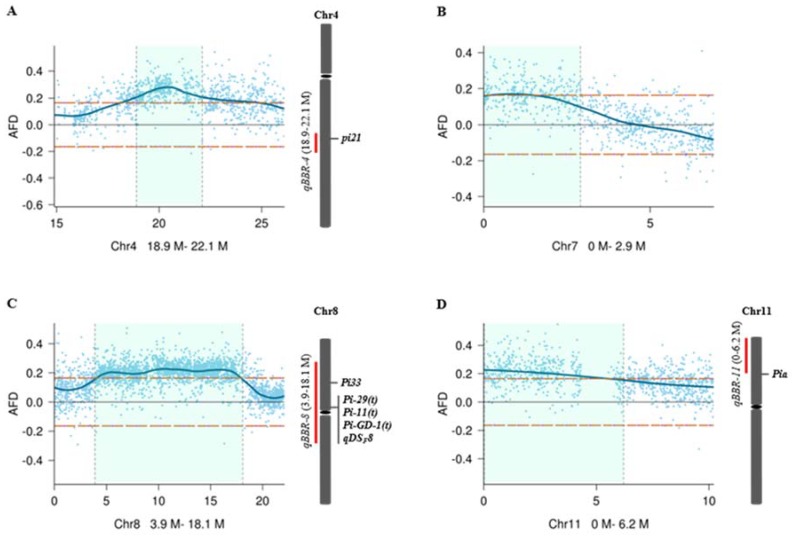
Estimates of confidence intervals of the four QTLs, *qBBR-4* (**A**), *qBBR-7* (**B**), *qBBR-8* (**C**), and *qBBR-11* (**D**). The horizontal orange dashed lines indicate the threshold (0.165) at the overall significance level of *p* < 0.05. The light green areas indicate 95% confidence intervals of the QTLs. Previously reported *R* genes or QTLs within or closely near the confidence intervals of *qBBR-4*, *qBBR-8*, and *qBBR-11* are indicated on the right. AFD: allele frequency difference.

Calculation results of the average allele frequency (AF) value for each marker showed that most of the identified SNP and short InDel markers had an expected AF value of around 0.5 in the four sub-pools ER-1, ER-2, ES-1, and ES-2 (Supplementary Figure S2), indicating no severe segregation distortion of the markers as a whole. Allele frequency difference (AFD) value between ER (ER-1 + ER-2) and ES (ES-1 + ES-2) pools was calculated, and four positive AFD peaks were detected in the fitted curve and exceeded the threshold (0.165 at the overall significance level of *p* < 0.05) (Figure 2A). Subsequently, unpaired t-tests were performed for the two replicated sub-pools for ER and ES. The *p*-values of each marker were estimated, and the peaks of negative logarithmic *p* value (NLP) (Figure 2B) were consistent with the peaks in AFD curve. These results suggest that there were four candidate QTLs located in these regions. The confidence intervals of the four QTLs located on chromosome 4, 7, 8, and 11 were estimated (Figure 2A and Figure 3, Table 2), and the four QTLs were named *qBBR-4*, *qBBR-7*, *qBBR-8*, and *qBBR-11*, respectively.

Heritability estimation showed that the additive heritability and dominance heritability of each QTL varied by 0.89%−2.09% and 0.01%−7.82%, respectively (Table 2). Among the four QTLs, *qBBR-4* had the largest effect, with the biggest additive heritability at about 2.09% (Table 2), suggesting that *qBBR-4* is a major QTL involved in basal resistance to blast disease.

### 2.4. Identification of a New Haplotype of pi21 as a Candidate Gene of qBBR-4

**Figure 4:**
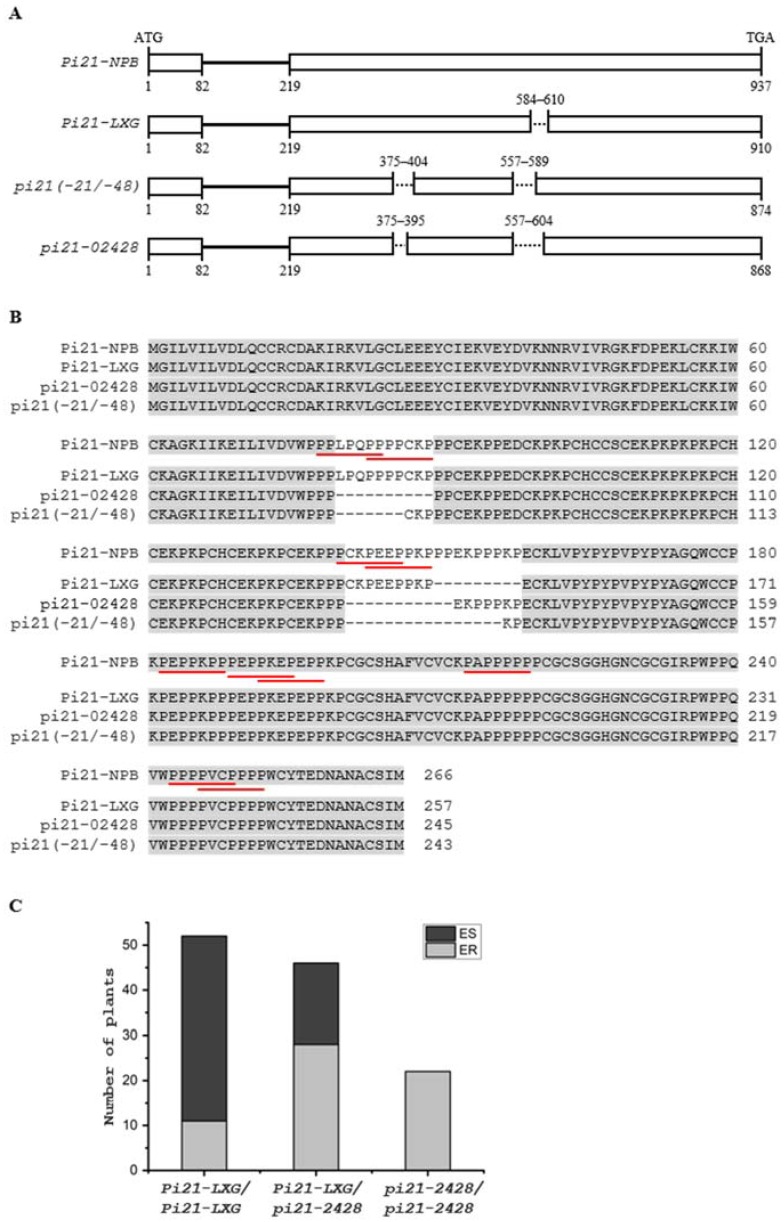
Identification of a novel haplotype of *pi21* as a candidate gene of *qBBR-4*. (**A**) Schematic diagram of the genomic coding region of the *Pi21* alleles in Nipponbare (*Pi21-NPB*), LXG (*Pi21-LXG*), and 02428 (*pi21-2428*), and the resistant allele of haplotype L containing double deletions of 21 bp and 48 bp (*pi21(-21/-48)*) [16]. Open boxes represent exons, lines represent introns, and dotted lines represent deletions. Numbers above the diagrams indicate positions of deletions corresponding to the *Pi21-NPB* allele, and numbers below the diagrams represent the start and end nucleotide positions of exons of each allele. (**B**) Alignment of the deduced amino acid sequences of Pi21-NPB, Pi21-LXG, pi21-2428, and pi21(-21/-48). Putative proline-rich motifs (PxxPxxP) for protein–protein interaction are underlined in red. (**C**) Genotype-phenotype correlation analysis of 61 ER individuals and 59 ES individuals. All the homozygous *pi21-2428* individuals belonged to the ER bulk.

To further refine candidate genes involved in basal resistance to blast disease, we searched previously reported *R* genes or QTLs within the confidence intervals of the four QTLs (Figure 3). While no previously reported *R* genes or QTLs were identified within or near the confidence interval of *qBBR-7* (Figure 3B), a previously identified *Pia* [26] gene was located near the confidence interval of *qBBR-11* (Figure 3D), and there were several *R* genes or QTL, including *Pi-11(t)* [27], *Pi-29(t)* [28], *Pi33* [29], *Pi-GD-1(t)* [30], and *qDS_F_8* [3], in the *qBBR-8* region (Figure 3C). Interestingly, *qBBR-4* was observed to be co-localized with a cloned recessive durable blast disease resistance QTL *pi21* (Figure 3A). *Pi21* has been found to have at least 12 variants (haplotypes A to L) based on InDel polymorphisms in the proline-rich region. While haplotype L containing double deletions of 21 bp and 48 bp (*pi21(-21/-48)*) resulting in deletions of the core motif “PxxPxxP” in the proline-rich region (Figure 4A) was identified, conferring durable blast disease resistance due to loss of function of *Pi21*, the other haplotypes carrying one of the two deletions or two smaller deletions did not confer high basal resistance to blast disease [16].

Sequence analysis of the *Pi21* alleles in LXG and 02428 showed that while the allele in LXG (*Pi21-LXG*) had a 27 bp deletion, the *pi21-2428* allele had double deletions of 30 bp and 33 bp, resulting in deletions of 10 aa and 11 aa of the core “PxxPxxP” motif, the same as in the *pi21(-21/-48)* allele (Figure 4A,B). Both *Pi21-LXG* and *pi21-2428* were different from the 12 identified haplotypes. Genotypic analysis of 61 individual ER and 59 individual ES plants showed that all *pi21-2428* homozygous seedlings showed resistance to blast disease. In contrast, about 79% (41 out of 52) of the *Pi21-LXG* homozygous, and 39% (18 out of 46) of *Pi21-LXG/pi21-2428* heterozygous seedlings showed susceptible to blast disease (Figure 4C). These results support *pi21-2428* as a candidate gene of *qBBR-4*, and suggest that while the 27 bp deletion did not affect *Pi21-LXG* function, double deletions of the 30 bp and 33 bp sequences could cause a defect in *pi21-2428* function, leading to high basal resistance to blast disease.

### 2.5. Assessment of the pi21-2428 Haplotype in 325 Rice Accessions

**Figure 5:**
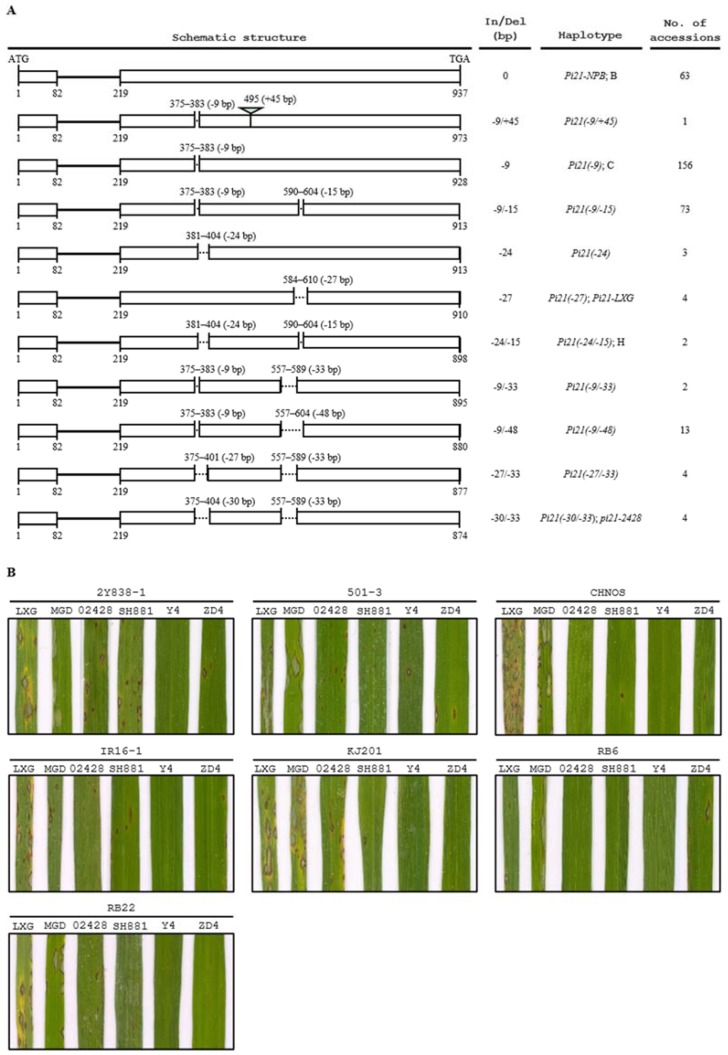
Assessment of haplotypes of *Pi21* in 325 rice accessions and blast inoculation of the *pi21-2428*-containing varieties. (**A**) A total of 11 haplotypes were identified among 325 rice accessions. The haplotypes were named based on insertion/deletion patterns. *Pi21-NPB*, *Pi21(-9)*, and *Pi21(-24/-15)* were identical to the previously identified haplotypes B, C, and H, respectively [16]. Open boxes represent exons, lines represent introns, and dotted lines represent deletions. Numbers above the diagrams indicate positions of deletions corresponding to the *Pi21-NPB* allele, and numbers below the diagrams represent the start and end nucleotide positions of exons of each allele. (**B**) Phenotypes of the *pi21-2428*-containing varieties 02428, ShuHui881 (SH881), Yong4 (Y4), and ZhengDa4Hao (ZD4) inoculated with *M. oryzae* isolates 2Y838-1, 501-3, CHNOS, IR16-1, KJ201, RB6, and RB22, respectively. Rice varieties LXG and MengGuDao (MGD) were used as highly susceptible controls.

Multiple sequence alignment of the *Pi21* alleles in 325 rice accessions revealed a total of 11 haplotypes among the tested accessions (Figure 5A, Supplementary Table S2). However, none of the accessions carried the resistant *pi21(-21/-48)* allele. The haplotypes were named based on insertion/deletion patterns. Among the 11 haplotypes, *Pi21-NPB* (the *Pi21* allele in Nipponbare), *Pi21(-9)*, and *Pi21(-24/-15)* were identical to the previously identified haplotypes B, C, and H, respectively (Figure 5A, Supplementary Table S2) [16], and the rest eight were novel types.

Among the 325 rice accessions, in addition to 02428, three Chinese *indica* varieties ShuHui881, Yong4, and ZhengDa4Hao were detected carrying the resistant *pi21-2428* allele. The *pi21-2428*-containing varieties were inoculated with seven *M. oryzae* isolates. As shown in Figure 5B, the two susceptible varieties LXG and MengGuDao (MGD) were highly susceptible to most of the *M. oryzae* isolates. The four *pi21-2428*-containing varieties showed complete resistance, moderate resistance, or moderate susceptibility to the isolates. For example, the *indica* variety ZhengDa4Hao was highly resistant to isolates KJ201 and RB22, and moderately resistant to isolates CHNOS and IR16-1, suggesting that there might be some *R* genes conferring the high resistance in ZhengDa4Hao against the four isolates. ZhengDa4Hao also showed susceptibility to isolates 2Y838-1, 501-3, and RB6, but the lesions on leaves were limited in size and much less than those on leaves of LXG or MGD. Overall, none of the *pi21-2428*-containing varieties showed highly susceptible to the seven *M. oryzae* isolates, suggesting that the four varieties possessed high basal resistance with significantly delayed and reduced development of disease lesions. 

## 3. Discussion

When compared with *R* gene-mediated, race-specific resistance, basal resistance has been presumed to be more durable [4,10]. Therefore, the identification of genes or QTLs conferring basal resistance is of significance in breeding crops with long-lasting resistance to plant diseases. In the present study, we employed BSA-Seq to identify four candidate QTLs *qBBR-4*, *qBBR-7*, *qBBR-8,* and *qBBR-11,* conferring basal resistance to rice blast disease on rice chromosomes 4, 7, 8, and 11, respectively. Building on advances in next-generation sequencing, the BSA-Seq method took advantage of pooled sequencing, which does not require the laborious process of genotyping of each individual from a large mapping population, allowing for rapid identification of candidate genes or QTLs controlling important agronomic traits [24,31].

Among the four QTLs detected in this study, *qBBR-4* had the largest additive effect (Table 2). The *qBBR-4* locus is located in a region of chromosome 4 with a known recessive blast disease resistant QTL *pi21,* identified in rice variety Owarihatamochi [16]. *Pi21* encodes a proline-rich protein, consisting of a putative heavy metal-binding domain and protein-protein interaction motifs. While the dominant *Pi21* appears to slow the plant’s defense responses, loss-of-function of *Pi21* (double deletions of 21 bp and 48 bp in the proline-rich region; haplotype L) confers durable resistance to blast disease in rice [16]. Sequence analysis revealed that the *pi21* allele in 02428, the parental line with high basal resistance, had double deletions of 30 bp and 33 bp, resulting in deletions of 10 aa and 11 aa in the proline-rich region, which house the core motif “PxxPxxP” (Figure 4A,B) for protein–protein interaction in multicellular organisms [16,32,33]. We further performed genotype-phenotype correlation analysis of 61 ER individuals and 59 ES individuals, and the results showed that all the homozygous *pi21-2428* individuals belonged to the ER bulk (Figure 4C). Taken together, these results suggest that double deletions of the 30 bp and 33 bp sequences of *Pi21* led to high basal resistance to blast disease, and that *pi21-2428* was the candidate gene of *qBBR-4*.

Previously, sequence analysis of the *Pi21* locus revealed 12 haplotypes (A to L) among 80 Asian cultivated rice varieties. Eleven of the haplotypes (A to K) carried an insertion or smaller deletions compared with the resistant *pi21(-21/-48)* allele, and did not confer resistance to blast disease [16]. In the present study, we detected a total of 11 haplotypes of *Pi21* among 325 rice accessions (Figure 5A, Supplementary Table S2), of which three were identical to the previously identified haplotypes B, C, and H [16], and eight were novel. Interestingly, the DNA variations of all the 20 detected haplotypes identified by Fukuoka et al. [16] and in this study were found to result in amino acid insertion/deletions, but not to cause premature termination of the predicted *Pi21* product, implying that the *Pi21* or *pi21* alleles maintain certain functions important for rice [34]. Besides 02428, we identified three more varieties, ShuHui881, Yong4, and ZhengDa4Hao, possessing the resistant *pi21-2428* allele. Inoculation testing with seven *M. oryzae* isolates indicated that, while the susceptible varieties LXG and MGD were highly susceptible to most of the *M. oryzae* isolates, the four *pi21-2428*-containing varieties showed complete resistance moderate resistance or moderate susceptibility to the *M. oryzae* isolates (Figure 5B). The results suggest that there should be some *R* genes other than *pi21-2428* conferring the complete or high resistance in the four *pi21-2428*-containing varieties. On the other hand, the moderately susceptible reactions with limited lesion size and number, to virulent *M. oryzae* isolates indicated that ShuHui881, Yong4, ZhengDa4Hao, as well as 02428, possessed high basal resistance to blast disease (Figure 5B). It is worth noting that while the resistant *pi21(-21/-48)* allele was found only in *japonica* rice [16], the three *pi21-2428*-containing varieties ShuHui881, Yong4, and ZhengDa4Hao were *indica* rice (Supplementary Table S2). Therefore, the varieties identified in the present study provide valuable resources for breeding rice varieties, especially *indica* varieties, with durable resistance to blast disease.

Transgressive segregation was observed in the F_2_ population of 02428 × LXG, where some F_2_ segregants showed more resistance than both parents (Figure 1B). This phenomenon implies that favorable alleles from both resistant and susceptible parents could be combined in the progeny, leading to a higher resistance than in the parents. In the present study, in addition to *pi21-2428*, we detected three other QTLs, *qBBR-7*, *qBBR-8*, and *qBBR-11*, on chromosomes 7, 8, and 11, respectively. Further identification and functional characterization of the three QTLs should be helpful to better understand the mechanisms underlying rice basal resistance to blast disease. Furthermore, the finding of transgressive segregation for blast resistance in the F_2_ population of 02428 × LXG indicates that pyramiding more basal resistance genes or QTL alleles with *pi21-2428* would be an effective approach to enhance durable resistance to rice blast disease [10,35].

## 4. Materials and Methods

### 4.1. Plant Materials and Blast Isolates

Rice cvs. 02428 and LXG were used to construct an F_2_ population to evaluate the segregation of blast disease reaction and for QTL mapping. Besides 02428 and LXG, a collection of 323 rice accessions consisting mainly of Chinese rice varieties or breeding materials were used for haplotype assessment in this study (Supplementary Table S2). The *M. oryzae* isolates CHNOS, Guy11, and KJ201 were kindly provided by Dr. Guo-Liang Wang (Department of Plant Pathology, Ohio State University, Ohio, USA), and isolates 2Y838-1, 501-3, IR16-1, RB6, and RB22 were collected from Fujian province, China.

### 4.2. Rice Blast Inoculations

Rice blast inoculations were carried out following a previously described spraying method [36]. Rice seedlings were grown in a greenhouse for about 12–14 days and were inoculated with *M. oryzae* spores at a concentration of 5 × 10^5^ spores mL^−1^. After inoculation, the seedlings were grown at 25 °C under high humidity for 4–5 days. Blast disease reactions were scored following a 0–5 scale (0–1: resistant, 2: moderately resistant, 3: moderately susceptible, 4–5: severely susceptible) [37].

### 4.3. Bulking, DNA Extraction, and Whole-Genome Resequencing

To understand the genetic basis of basal resistance to rice blast disease in 02428, a total of 626 F_2_ plants derived from a cross between 02428 and LXG were inoculated by RB22, and were investigated for the segregation of disease reaction. For bulked segregant analysis, about 10,000 individuals of the F_2_ population of 02428 × LXG were inoculated with the *M. oryzae* isolate RB22. About 126 highly resistant and 120 highly susceptible F_2_ individuals were screened to generate the ER and ES bulks, respectively. Both the ER and ES bulks were divided into two replicates, ER-1 (56 individuals) and ER-2 (70 individuals) for the ER bulk, and ES-1 (60 individuals) and ES-2 (60 individuals) for the ES bulk. The genomic DNAs of the four bulked samples were extracted and were mixed with equal amounts. DNA samples of the two parents, 02428 and LXG, and the four pools were subjected to whole-genome resequencing using the Illumina HiSeq X Ten platform, followed by standard paired-end 150 bp sequencing library construction protocols.

### 4.4. Analysis of Reads and Variants

The raw reads were cleaned and trimmed using BBDuk program of BBTools (http://jgi.doe.gov/data-and-tools/bbtools/). The paired reads were mapped to the IRGSP-1.0 reference rice genome (http://rapdb.dna.affrc.go.jp) by using Burrows-Wheeler Aligner based on the Maximal Exact Matches algorithm (BWA MEM) and the alignments were processed by SAMTools [38,39,40]. Freebayes was used to call SNPs and InDels, with default parameters [41]. To obtain reliable polymorphic markers, variant filtering was performed by custom perl scripts: firstly, SNPs or short InDels exhibiting polymorphism between the two parents were screened; secondly, to further avoid severe segregation distortion, SNPs or short InDels with AF values from 0.3 to 0.7 were retained. These markers were annotated by snpEff [42].

### 4.5. QTL Analysis

The marker set was employed to map QTLs. The replicated sub-pools ER-1, ER-2, and ES-1, ES-2 were firstly incorporated into one ER pool and one ES pool, respectively. AFD value between the ER and ES pools was calculated and then smoothed by block regression, following the Block Regression Mapping methodology [43]. The block size used for the regression was set to be 20 kb. The AFD curve threshold at the overall significance level of 0.05 was estimated under the assumption of theoretical allele frequency (= 0.5) in the F_2_ population. For each significant AFD peak (candidate QTL), the 95% confidence interval was estimated. In addition, unpaired t-tests based on the two biological replicates of the ER and ES pools were performed to validate the candidate QTLs following the X-QTL-seq method [44]. According to the peak AFD value, the heritability of each QTL was estimated using the method of Pooled QTL Heritability Estimator (PQHE) [45].

### 4.6. Detection of Haplotypes of Pi21

Genomic DNAs of the rice accessions (Supplementary Table S2) were subjected to genotyping for haplotypes of *Pi21*. PCR products were amplified with primers P21-F (5′-CAAGGCTAATCAGCAGTGT-3′) and P21-R (5′-TTGGCGTTGTCCTCGGTGT-3′). DNA sequences of PCR products were aligned using Clustal W [46].

## Figures and Tables

**Table 1:** Chromosome-wise distribution of the identified single-nucleotide polymorphisms (SNPs) and short InDels.

Chr.	Length	SNP		Short InDel	
		Number	Density (per kb)	Number	Density (per kb)
Chr1	43,270,923	48,669	1.12	7735	0.18
Chr2	35,937,250	67,302	1.87	9069	0.25
Chr3	36,413,819	20,399	0.56	3336	0.09
Chr4	35,502,694	42,156	1.19	5558	0.16
Chr5	29,958,434	67,386	2.25	9189	0.31
Chr6	31,248,787	34,848	1.12	5309	0.17
Chr7	29,697,621	25,681	0.86	4040	0.14
Chr8	28,443,022	56,474	1.99	7152	0.25
Chr9	23,012,720	23,331	1.01	2965	0.13
Chr10	23,207,287	36,435	1.57	4062	0.18
Chr11	29,021,106	31,309	1.08	4680	0.16
Chr12	27,531,856	15,522	0.56	2671	0.10
Total	373,245,519	469,512	1.26	65,766	0.18

**Table 2:** Estimates of position and heritability of the four identified QTLs.

QTL	Chr.	AFD value ^a^	Pos. (Mb) ^b^	Interval (Mp) ^c^	Max. NLP ^d^	$h_{A}^{2}$ (%) ^e^	$h_{D}^{2}$ (%) ^f^
*qBBR-4*	4	0.278	20.46	18.90–22.10	0.90	2.09	7.82
*qBBR-7*	7	0.172	0.86	0–2.91	0.65	0.89	0.26
*qBBR-8*	8	0.227	10.63	3.89–18.09	0.85	1.56	0.01
*qBBR-11*	11	0.226	0.02	0–6.21	0.73	1.54	3.50

^a^ Maximum value of the peak of the AFD curve; ^b^ Chromosome position of the peak of the AFD curve; ^c^ Estimated based on the 95% confidence; ^d^ The most significant *p*-value of the peak, which was converted to negative logarithmic *p* (NLP) value; ^e^ Heritability attributed to additive effect of the QTL; ^f^ Heritability attributed to dominance effect of the QTL.

## Supplementary Materials

Supplementary materials can be found at https://www.mdpi.com/1422-0067/21/6/2162/s1.
