# Supplementary material for: Bulked Segregant Analysis Coupled with Whole-Genome Sequencing (BSA-Seq) Mapping Identifies a Novel pi21 Haplotype Conferring Basal Resistance to Rice Blast Disease
Source: Int J Mol Sci. 2020 Mar 21;21(6):2162. doi: 10.3390/ijms21062162 (PMC7139700; doi:10.3390/ijms21062162)
Supplement: Supplementary file 1 [file ijms-21-02162-s001.zip › Supplementary Figure S2-2019.12.06.pptx]

## Slide 1
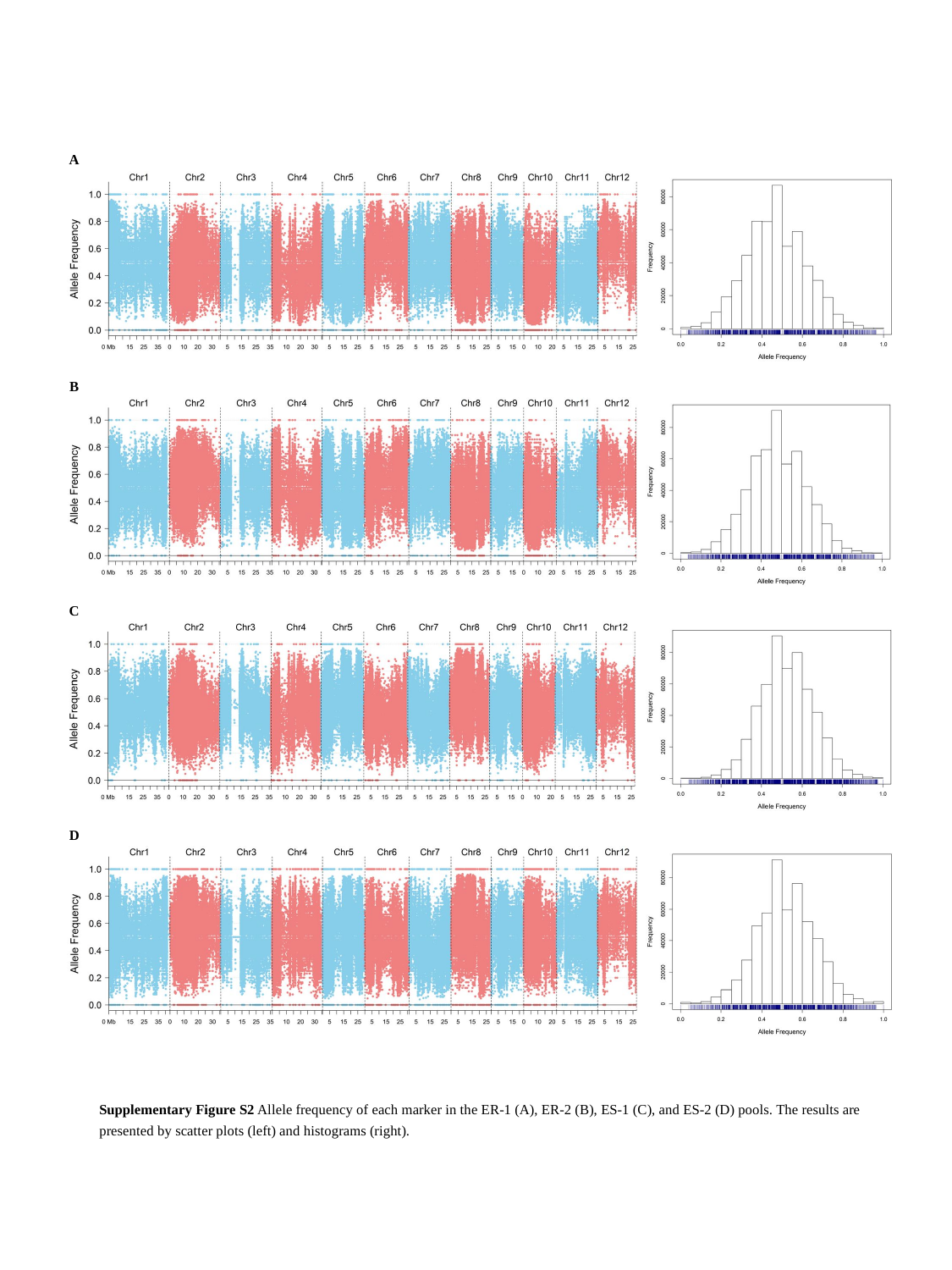

A
B
C
D
Supplementary Figure S2 Allele frequency of each marker in the ER-1 (A), ER-2 (B), ES-1 (C), and ES-2 (D) pools. The results are presented by scatter plots (left) and histograms (right).
