# Supplementary material for: Bulked Segregant Analysis Coupled with Whole-Genome Sequencing (BSA-Seq) Mapping Identifies a Novel pi21 Haplotype Conferring Basal Resistance to Rice Blast Disease
Source: Int J Mol Sci. 2020 Mar 21;21(6):2162. doi: 10.3390/ijms21062162 (PMC7139700; doi:10.3390/ijms21062162)
Supplement: Supplementary file 1 [file ijms-21-02162-s001.zip › Supplementary Table S1-2019.12.06.docx]

**Supplementary Table S1** Summary of sequencing and reads mapping

| Sample | Materials | Total reads | Depth | Mapping rate |
| --- | --- | --- | --- | --- |
| 02428 | 15 individuals | 118,663,740 | 43.08× | 98.45% |
| LXG | 15 individuals | 95,251,874 | 29.14× | 96.74% |
| ER-1 | 56 extremely resistant F_2_ progenies | 90,744,296 | 27.96× | 97.37% |
| ER-2 | 70 extremely resistant F_2_ progenies | 97,849,554 | 29.42× | 97.61% |
| ES-1 | 60 extremely sensitive F_2_ progenies | 158,943,276 | 46.14× | 95.35% |
| ES-2 | 60 extremely sensitive F_2_ progenies | 91,185,142 | 28.53× | 93.43% |

Note: Depth, average per-base coverage depth of mapped reads. Mapping rate, mapped reads divided by total reads.
