# Supplementary material for: Bulked Segregant Analysis Coupled with Whole-Genome Sequencing (BSA-Seq) Mapping Identifies a Novel pi21 Haplotype Conferring Basal Resistance to Rice Blast Disease
Source: Int J Mol Sci. 2020 Mar 21;21(6):2162. doi: 10.3390/ijms21062162 (PMC7139700; doi:10.3390/ijms21062162)
Supplement: Supplementary file 1 [file ijms-21-02162-s001.zip › Supplementary Figure S1-2019.12.06.pptx]

## Slide 1
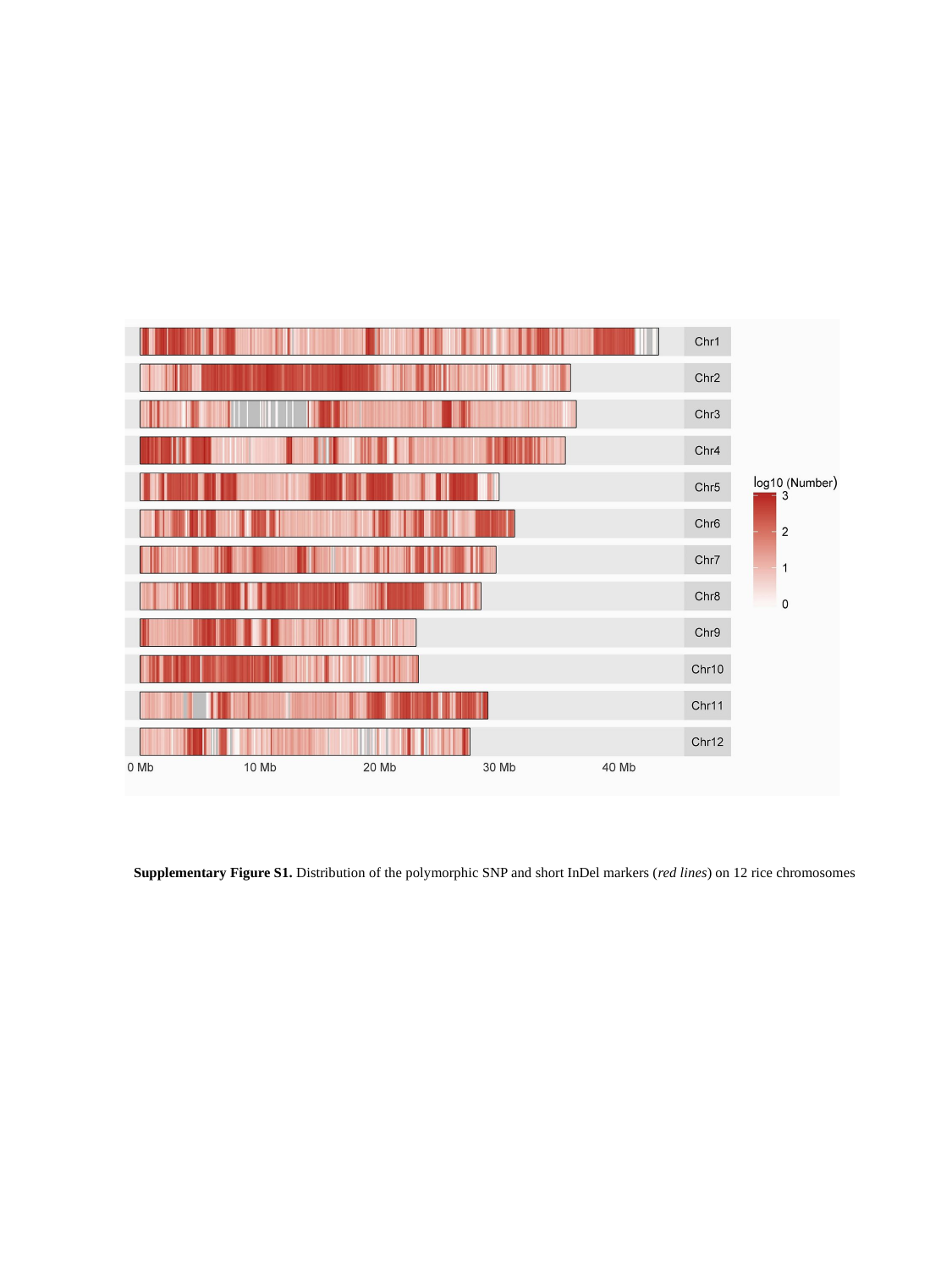

Supplementary Figure S1. Distribution of the polymorphic SNP and short InDel markers (red lines) on 12 rice chromosomes
