# Supplementary material for: Bulked Segregant Analysis Coupled with Whole-Genome Sequencing (BSA-Seq) Mapping Identifies a Novel pi21 Haplotype Conferring Basal Resistance to Rice Blast Disease
Source: Int J Mol Sci. 2020 Mar 21;21(6):2162. doi: 10.3390/ijms21062162 (PMC7139700; doi:10.3390/ijms21062162)
Supplement: Supplementary file 1 [file ijms-21-02162-s001.zip › Supplementary Table S2-2019.12.06.doc]

**Supplementary Table S2** Assessment of the variations of *Pi21* in 02428, LiXinGeng (LXG), and 323 rice accessions

| **Haplotype^a^** | **Origin** | **Subspecies** | **Cultivars or breeding materials** | **No. of cultivars** | **Total** |
| --- | --- | --- | --- | --- | --- |
| *Pi21-NPB;*  Haplotype B | China | *Indica* | 710S, ChengHui, ChiShuHui, ChuanHui448, CY85-43, EnHui99-64, GuangHui, JinYouZao48, LongHui, MiYang23, P03094, PeiC122, SanHuangZhan, WCR1, WuHui, XiaoJiuGu, Zhe733, ShuHui527 | 18 | 63 |
|  |  | *Japonica* | BaiZhuGeng, C418-11, ChunJiang101, ChunJiang110, DN710, G49, G51, G56, HaiLinYiHao, HuaGeng3Hao, Jia58, Jia66, JiaHe228, JiaHe236, KongYu131, LongGeng31, LunHui422, LuYu132, MengGuDao, NanGeng5002, R109, R187Geng, SongGeng9, TaiKen9, TaiNong5, TaiNong67, XiuShui121, XiuShui134, XiuShui519, Yan8, Yong15, YunYin, ZheGeng41, ZheGeng99, ZhongHua11Hao | 35 |  |
|  | International areas | *Indica* | DP4, CS651, IR24, IR64, IR661-1, N22, Pokkali | 7 |  |
|  |  | *Japonica* | Kitaake, Koshihikari, UNBONG8 | 3 |  |
| *Pi21(-9/+45)* | China | *Indica* |  | 0 | 1 |
|  |  | *Japonica* |  | 0 |  |
|  | International areas | *Indica* | IR6560-126-2-B | 1 |  |
|  |  | *Japonica* |  | 0 |  |
| *Pi21(-9);*  Haplotype C | China | *Indica* | 75-1-107, 143, 7055, YueFengB, BiWuSheng, Ce64, Ce148, ChengHui707, D62B, D702B, DaZiLaiShan, DiXiangB, DuoHui43, Duoxi1Hao, DX33, FuHui7018, FuHui7185, Gu154, GuangHui998, GuangLuAi4Hao, Gui32, GuiChao2Hao, GuiSiXiangNuo, HangHui57, Hang1Hao, HaoLai, HongChaXiaZhi, HongGengHanGu, HuaGuJiangLai, HuaXiangB, HuBeiXiangDao, Hui68, Hui84, Hui207, Hui397, JiangYouHui151, JiaYu164, JiaYu293, JiaYu948, JingNuo6Hao, JinXiBai, ZaoHui1Hao, L301B, LeHui188, Lmb13-1, Lmb24-1, Lmb51-1, Lmb89-2, MinBeiWanXian, MingHui63, MingHui72, MingHui77, MingHui81, MingHui86, MingHui2155, MinHui3119, MoWangGuNeiZa, NanHui511, NanTianGangJiu, NeiHui99-14, QiBaiTou, QingKe, R02w05, R157, R178, R524, R1128, R1259, R2115, R3203, RK007, SanLiCun, SE21S, ShuHui498, ShuHui881, T230, TaiFengB, TaiZhongZaiLai1, TQ347, W31, WuJuHongGu, WuKeZhan, WuHui898, XiangDao, XinFengZhan1, YangDao4, YanShuiChi, YiXianHong, You1B, ZaoHui63, ZheHui (J1-99), ZhengDa36, ZhengDa86, ZhengDa109, ZhengDa116, ZhengDa118, ZhengDa119, ZhengDa131, ZhengDa133, ZhengDa146, ZhengDa219, ZhengDa3119, ZhenShan97B, Zhi1, Zhi7, ZhongJian100, ZhongShiB, ZhongYu1, ZhuZhenB, ZiNuo, ZR21, ZR30, ZR137 | 113 | 156 |
|  |  | *Japonica* | GanGanNuo, HuHui628, NianKeNuo, ShuiYuan290, ShuiYuan337, Yong1, Yong2, Yong3, Yong12 | 9 |  |
|  | International areas | *Indica* | 96IR-49, IR0401, IR0404, IR0405, IR0406, IR0407, IR0410, IR0411, IR0413, IR0415, IR0416, IR0417, IR0420, IR0424, IR0425, IR0427, IR0429-1, IR0432-1, IR0459, IR661-1, IR1469-1, IR04553, IR04557, IR13427-60-1-3-2-2, IR55433-63, IR68903B, IR72158-16-16-3-3, IR72164-368-6-2-2-2, IR74642-195-1-3-2, JN-2, MR-1, NDR96, R900 | 33 |  |
|  |  | *Japonica* | Balilla | 1 |  |
| *Pi21(-9/-15)* | China | *Indica* | 618B, 629B, II-32B, AiJiaoNanTe, AiTuoGu151, BaiKeHuaLuo, BaoXie123B, BoBaiB, C712001, ChuanXiang29B, D8, D297B, DianLong, ErJiuNan1Hao, EZao11, Feng008B, FengAiZhan2, FuHui408, FuXiang, G8B, GD-5S, GS, GuangKang13B, GuFengB, H359, HanMaDao, HD9802S, HuangHuaZhan, HuaZhan, HuiFengB, Jiabala, JiangNongZao1Hao, JiaoShuiZao, Jin23B, KB-1, KB-2, Liusha1Hao, LongTePuB, M76B, Min2B, NanTeHao, NanYouZhan, P03092, Shen95B, T55B, TianFengB, V20B, XiangWanXian1Hao, XiangZaoXian7Hao, YaZhan, YiB-1, YueNongSiMiao, ZaoGangB, ZhaoYang1B, ZheFu802, ZhenXian232, ZhongJiuB, ZhongZao23 | 58 | 73 |
|  |  | *Japonica* | BaiMangGeng, BaiMaoDao, DN730, XingGuo | 4 |  |
|  | International areas | *Indica* | C39, CP112, Da224, GIZA176, IR661-1, IR58025B-2, IR688998B, IR76928-74-3-2-1, IR88988B, BL123 | 10 |  |
|  |  | *Japonica* | Katy | 1 |  |
| *Pi21(-24)* | China | *Indica* | TeQingXuan, XiangHui91269 | 2 | 3 |
|  |  | *Japonica* |  | 0 |  |
|  | International areas | *Indica* | IR0435 | 1 |  |
|  |  | *Japonica* |  | 0 |  |
| *Pi21(-27); Pi21-LXG* | China | *Indica* | HuNanNuo, LaoHongGu, YeWeiPan | 3 | 4 |
|  |  | *Japonica* | LiXinGeng (LXG) | 1 |  |
|  | International areas | *Indica* |  | 0 |  |
|  |  | *Japonica* |  | 0 |  |
| *Pi21(-24/-15);*  Haplotype H | China | *Indica* |  | 0 | 2 |
|  |  | *Japonica* |  | 0 |  |
|  | International areas | *Indica* | IR0414-1, IR04558 | 2 |  |
|  |  | *Japonica* |  | 0 |  |
| *Pi21(-9/-33)* | China | *Indica* | MeiHuaNuo, WuHongXian | 2 | 2 |
|  |  | *Japonica* |  | 0 |  |
|  | International areas | *Indica* |  | 0 |  |
|  |  | *Japonica* |  | 0 |  |
| *Pi21(-9/-48)* | China | *Indica* | BaiXiangHe, FuHui718, FuHui838, Gui630, LaoLaiHuang, LuHui17, TaiYou, ZhongXiang1Hao | 8 | 13 |
|  |  | *Japonica* |  | 0 |  |
|  | International areas | *Indica* | IR0412, IR0452, IR04552, IR0464, IR73459-120-2-2-3-1 | 5 |  |
|  |  | *Japonica* |  | 0 |  |
| *Pi21(-27/-33)* | China | *Indica* |  | 0 | 4 |
|  |  | *Japonica* |  | 0 |  |
|  | International areas | *Indica* | IR0408-1, IR0462, IR0472J2, IR95-3 | 4 |  |
|  |  | *Japonica* |  | 0 |  |
| *Pi21(-30/-33); pi21-02428* | China | *Indica* | ShuHui881, Yong4, ZhengDa4Hao | 3 | 4 |
|  |  | *Japonica* | 02428 | 1 |  |
|  | International areas | *Indica* |  | 0 |  |
|  |  | *Japonica* |  | 0 |  |
| Total |  | | | | 325 |
|  | China | *Indica* |  | 206 | 257 |
|  |  | *Japonica* |  | 51 |  |
|  | International  areas | *Indica* |  | 63 | 68 |
|  |  | *Japonica* |  | 5 |  |

^a^ A total of 11 haplotypes of *Pi21* were identified among 325 rice accessions; The haplotypes were named based on insertion/deletion patterns. Haplotypes *Pi21-NPB* (the *Pi21* allele in Nipponbare), *Pi21(-9)*, and *Pi21(-24/-15)* were identical to the previously identified haplotypes B, C, and H, respectively (Fukuoka et al. 2009). Haplotypes *Pi21(-27)* and *Pi21(-30/-33)* were named *Pi21-LXG* and *pi21-02428*, respectively.
